# Integrated System of Structural Health Monitoring and Intelligent Management for a Cable-Stayed Bridge

**DOI:** 10.1155/2014/689471

**Published:** 2014-07-21

**Authors:** Bin Chen, Xu Wang, Dezhang Sun, Xu Xie

**Affiliations:** ^1^College of Civil Engineering and Architecture, Zhejiang University, Hangzhou 310058, China; ^2^State Key Laboratory Breeding Base of Mountain Bridge and Tunnel Engineering, Chongqing Jiaotong University, Chongqing 400074, China; ^3^Institute of Engineering Mechanics, China Earthquake Administration, Harbin 150080, China

## Abstract

It is essential to construct structural health monitoring systems for large important bridges. Zhijiang Bridge is a cable-stayed bridge that was built recently over the Hangzhou Qiantang River (the largest river in Zhejiang Province). The length of Zhijiang Bridge is 478 m, which comprises an arched twin-tower space and a twin-cable plane structure. As an example, the present study describes the integrated system of structural health monitoring and intelligent management for Zhijiang Bridge, which comprises an information acquisition system, data management system, evaluation and decision-making system, and application service system. The monitoring components include the working environment of the bridge and various factors that affect bridge safety, such as the stress and strain of the main bridge structure, vibration, cable force, temperature, and wind speed. In addition, the integrated system includes a forecasting and decision-making module for real-time online evaluation, which provides warnings and makes decisions based on the monitoring information. From this, the monitoring information, evaluation results, maintenance decisions, and warning information can be input simultaneously into the bridge monitoring center and traffic emergency center to share the monitoring data, thereby facilitating evaluations and decision making using the system.

## 1. Introduction

In recent years, the designs of bridges have tended to become more flexible, where their form and function are increasingly complex. Thus, guaranteeing the safety of these bridge structures is an important issue. Safety monitoring is expensive but it is not possible to guarantee the safety of a bridge based only on inspections and maintenance. At present, structural health monitoring (SHM) techniques are becoming important for guaranteeing the safety of bridge structures, especially for the large-span bridge structures. Thus, SHM techniques are key research areas in the academic and engineering domains [1]. SHM techniques have been developed extensively and various mature technologies are in use in large-span bridges, including the Hakucho Bridge in Japan [2], Bill Emerson Memorial Bridge in the USA [3], Jindo Bridge in South Korea [4], Tsing Ma Bridge and Ting Kau Bridge in Hong Kong [5], and Sutong Bridge and Jiangyin Changjiang River Bridge in China. These systems guarantee the safe operation of the bridge and the life spans of bridges are extended using various methods [6–9]. At the same time, through finding the damages of bridge timely, the cost of maintenance can be reduced considerably and the losses due to traffic closures during active maintenance can also be avoided [10]. In recent years, the application of devices such as wireless sensors and GPS [11, 12] to large-span bridge health monitoring has addressed the problem of inconvenient wired sensor placement and facilitated the construction of SHM systems and the long-term monitoring of large-span bridges [13–16].

In the present study, the integrated system of structural health monitoring and intelligent management of Zhijiang Bridge in Hangzhou City, China, is used as an example to illustrate the overall system and the four functional sub-systems used in a SHM, that is, an information acquisition system (IAS), data management system (DMS), evaluation and decision making system (EDS), and application service system (ASS). This study provides a reference to facilitate the construction of SHM systems for other bridges.

## 2. Bridge Description

**Figure 1:**
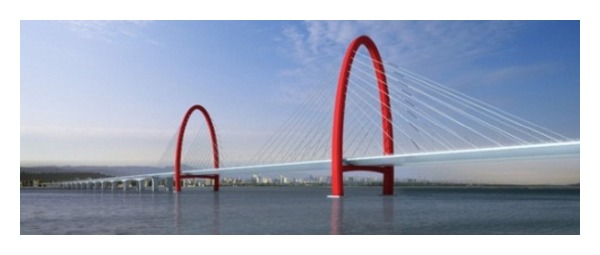
Photograph of Zhijiang Bridge.

Zhijiang Bridge is located in Hangzhou, China, where it crosses the Qian Tang River and it is a two-arched tower and twin-plane cable-stayed bridge (Figure 1), where the combined span is 116 + 246 + 116 = 478 m. The bridge structure employs a half-floated system. The bridge tower is an arched steel structure where the central axis has an elliptical curve, the height of the tower is 90.5 m, the central width of the tower is 44.4 m, and the width of the pylon in the lateral direction is 3.6 m. The pylon exhibits linear variation up and down in the vertical direction, where the width at the top of the tower is 4.0 m and the width at the bottom of the tower is 6.0 m. Apart from the slabs around the tower section, it was designed as two webs in the vertical direction and it comprises a single-box/three-cell section. The distance between the transverse diaphragms in vertical direction is ca 1.75–2.0 m. The transverse diaphragm that corresponds to the panel on the horizontal beam of the tower was designed in the horizontal direction, whereas the other transverse diaphragms were designed in the vertical direction. The beam of the main bridge is a streamlined steel box girder, which is single-box and multiple-cell, where the two sides comprise wind fairings and sideways, the height of the beam is 3.5 m (the outer size of the box), the full width is 41.36 m (including the wind fairing), and the thickness of the top slab is 16 mm. There were totally 88 cables in the bridge, which consists of epoxy coating unbounded steel strand. The top of the cable was anchored at the anchor box of tower and the bottom was anchored at the steel anchor box of main beam.

## 3. Bridge Monitoring System

Zhijiang Bridge is a two-arched tower and cable-stayed bridge, which has the largest span, the highest steel arch tower, and the widest bridge floor of this type of bridge. The main characteristics of the bridge include the complex structural system and mechanical properties, the design and construction of the arched steel tower lack technical standards, the high variable range of the cable-stayed angle, and the connections between the steel tower and the pile cap are anchored by bolts. Thus, it was necessary to build a SHM system for Zhijiang Bridge to meet the operational management needs, improve the level of prealarm security, enhance the efficiency of maintenance management, and facilitate scientific and effective operational management. The core tasks of the SHM system are to determine the environmental load, structural response, partial damage, and other information, as well as to obtain security state information for the traffic and structure based on a comprehensive assessment of this information, thereby ensuring the structural safety and efficient and economic operational decision-making to provide complete technical support.

The integrated system of structural health monitoring and intelligent management used by Zhijiang Bridge comprises four functional sub-systems, that is, IAS, DMS, EDS, and ASS. IAS is a lower level system that includes a data monitoring subsystem and maintenance management subsystem. DMS and EDS are middle level systems, where the former includes a data management subsystem and the latter includes a structural state evaluation subsystem and a maintenance management and security prealarming subsystem. ASS is the upper level system that includes a user interface subsystem. Using wired fiber communication and direct inputs, the IAS exchanges data with the DMS according to acquisition rules. In addition to the summarizing, filing, storage, and management of data, the DMS also provides necessary data support for the EDS, as well as providing data queries for the ASS. The structural state evaluation subsystem of the EDS provides analytical results that facilitate decision making and prealarming maintenance management, where the security prealarming subsystem feeds back to the ASS.

**Figure 2:**
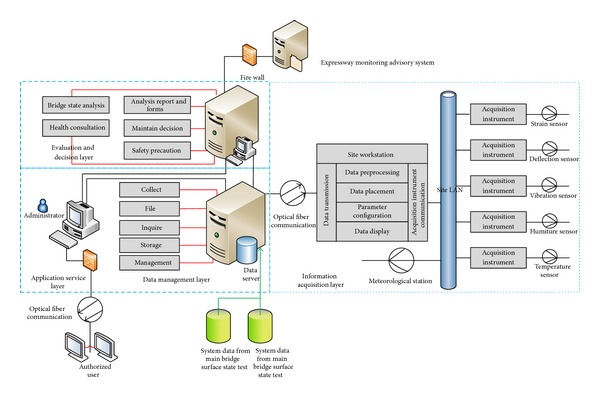
Overall workflow of the system.

Figure 2 shows the overall workflow of the integrated system of Zhijiang Bridge. The DMS and EDS form the interface with the Expressway Monitoring Advisory System (EMAS) of Hangzhou City. The monitoring information, evaluation results, maintenance decisions, and warning information related to the bridge can be input simultaneously into the monitoring center of Zhijiang Bridge and the Traffic Emergency Command Center of Hangzhou City, thereby sharing the monitoring data and decisions generated by the system.

### 3.1. Design and Implementation of the IAS

#### 3.1.1. Sensor Systems

**Figure 3:**
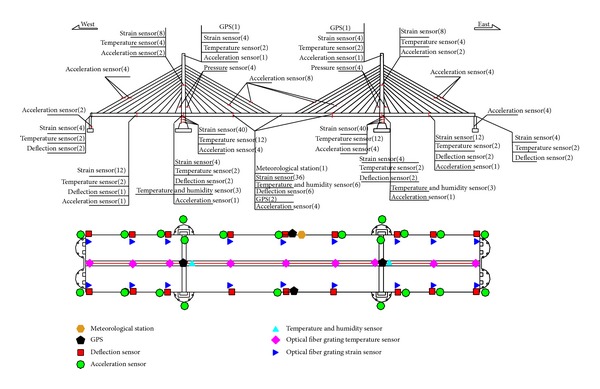
Layout of sensors on Zhijiang Bridge.

Depending on the aims of SHM, the sensor module design mainly comprises the sensor type, monitoring positions, and the number of the monitoring point. The sensor module in the data monitoring subsystem of the SHM system used by Zhijiang Bridge includes work environment monitoring (wind load, environmental temperature, humidity, rainfall, visibility, temperature and humidity of the steel box girder and steel arch tower, and vehicle load), structure spatial deformation monitoring, bridge alignment monitoring, section stress monitoring, fatigue and welding crack monitoring, and vibration monitoring in the steel arch tower and steel box girder, impact force monitoring in the bridge pier, earthquake response monitoring, cable force monitoring, and anchor force monitoring in the steel-concrete joint segment. The numbers of sensors and their layout are shown in Figure 3.

*Work Environment Monitoring*

(1)* Monitoring the Wind Load, Environmental Temperature, Humidity, Rainfall, and Visibility*. The middle span of Zhijiang Bridge is obviously affected by the wind, where the variation in the internal force is greatest during simulations where the system temperature increases and decreases. Thus, there are monitoring points for the wind load, environmental temperature, and humidity on the middle main span of the bridge. The monitoring instrument is fixed to the bridge floor of the steel box girder on the downstream side of the main span of the main bridge via a stainless steel column, which is located on the outside of a side guardrail.

**Figure 4:**
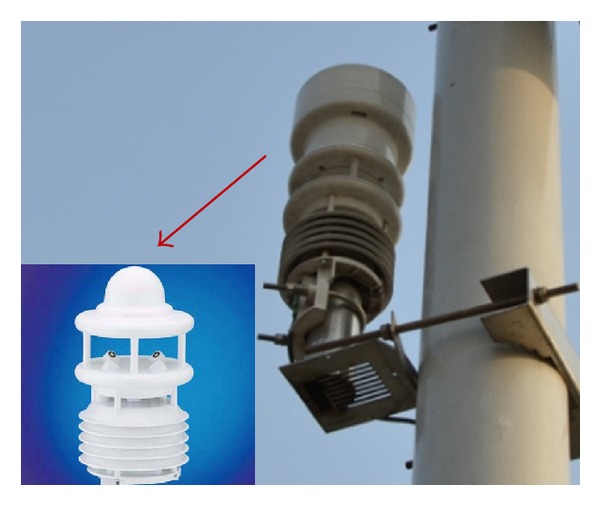
Meteorological station.

**Figure 5:**
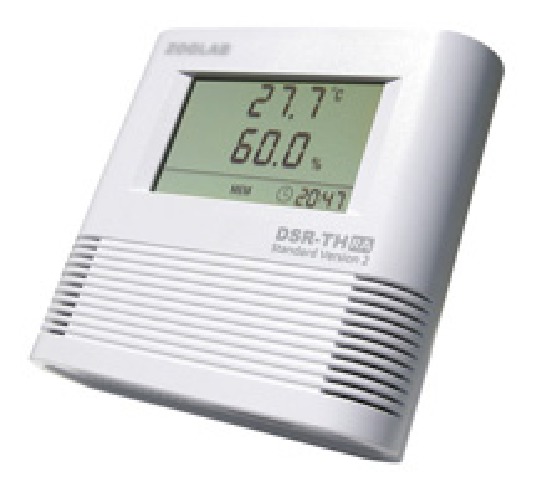
Hygrothermograph.

A professional meteorological station (Lufft) is used to monitor the atmospheric temperature and humidity, visibility, wind speed and direction, and rainfall.  Figure 4 and Table 1 show images and the technical specifications of the instrument, respectively.

(2)* Temperature and Humidity Monitoring on the Steel Box Girder and Steel Arch Tower.* The structural components of the main Zhijiang Bridge are steel box girders and steel arch towers. The environmental temperature and humidity is high around the bridge site, so the steel box girders and steel arch towers are readily corroded. A ventilation and dehumidification system is present inside the steel structure of the bridge, which can control the temperature and relative humidity inside the steel box girder and steel arch tower. However, in extreme high temperature or extreme high humidity weather conditions, or during sudden power failures, the humidity will increase inside the steel structure, which will affect the durability of the steel structure. Therefore, a temperature and humidity sensor is placed in an appropriate position inside the steel box girders and the steel arch towers, which monitor the changes in temperature and humidity. Based on the structural characteristics of the steel box girders and the steel arch towers, two sections of steel box girder and four sections of steel arch tower at the junction of steel box girder and steel arch tower have a hygrothermograph. A networked-edition DSR temperature and humidity recorder is used to monitor the temperature and humidity of the steel box girder and steel arch tower, a photograph of which is shown in Figure 5 and the main technical specifications are presented in Table 2.

(3)* Vehicle Load Monitoring.* Vehicle load monitoring must be performed in real-time and the accurate axle loads of passing vehicles need to be determined in a dynamic state, including calculating the vehicle weight and obtaining the license plate numbers of overweight vehicles, which are used to assist the management and control of overweight vehicles by the bridge management department. Therefore, a Weigh-in-Motion system with a video capture function is used to monitor the vehicle load. The bridge is bidirectional with six lanes, so the Weigh-in-Motion system needs to monitor six points in total.

The Weigh-in-Motion system used to monitor the traffic load is manufactured by TDC Systems (UK), which is a well-known producer of international instruments for intelligent traffic monitoring. The main instrument parameters are shown in Table 3.

**Figure 6:**
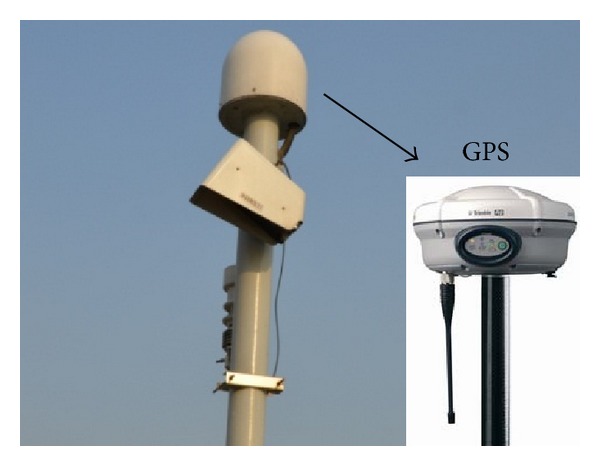
GPS sensor.

*Spatial Structure Deformation Monitoring.* Finite elements analysis of Zhijiang Bridge showed that the spatial deformation in the top of the steel arch tower and steel box girder in the middle main span was the largest with different load combinations. Thus, a Global Position System (GPS) is used to monitor the spatial deformation at these sites. Two GPS sensors are positioned at the top of the east and west steel towers, and two GPS sensors are located upstream and downstream of the steel box girder in the middle main span. GPS (Trimble, USA) sensors are used for spatial deformation monitoring on the steel arch towers and steel box girders on Zhijiang Bridge (Figure 6). The same brand of GPS sensor is used to monitor a number of major projects in China, such as Hangzhou Bay Bridge, which is the longest ocean-crossing bridge in the world. The main technical specifications of the GPS sensors are shown in Table 4.

**Figure 7:**
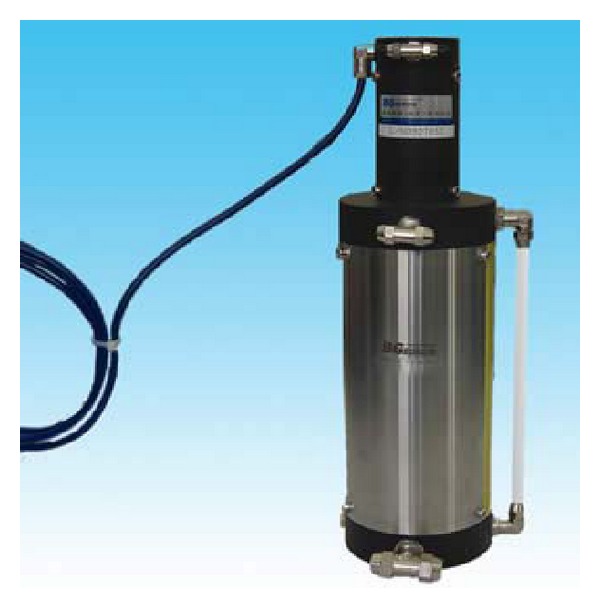
Hydrostatic level gauge.

*Bridge Alignment Monitoring.* The positions with the maximum variation in the displacement of the main beam with various load combinations are the middle main span and the middle side span, and the variation in the displacement of the quarter-point of the main span is also high. Thus, these are all important points for monitoring the alignment of Zhijiang Bridge. Subsidence points on the pier tops are also monitored in addition to monitoring the alignment of the main bridge network. The east approach of Zhijiang Bridge is a continuous beam of 60 + 11 × 86 + 60 m, which is as important as the main bridge in terms of the risk of damage. A three-span structure of the main bridge on the Dongfei navigation channel is used as the key monitoring point, where alignment monitoring sensors are positioned in the middle span of the approach and on the pier tops on both sides of the main span. Based on the characteristics of Zhijiang Bridge, as well as economic, convenience, stability and durability considerations, a hydrostatic level gauge is used for alignment monitoring (Figure 7) and its technical specification are shown in Table 5.

**Figure 8:**
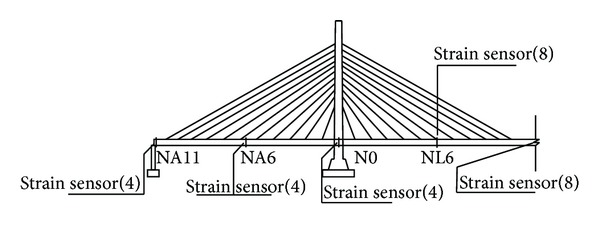
Stress and fatigue monitoring points on the box girder.

**Figure 9:**
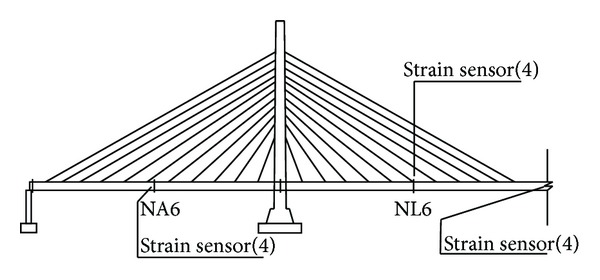
Welding crack monitoring points on the box girder.

**Figure 10:**
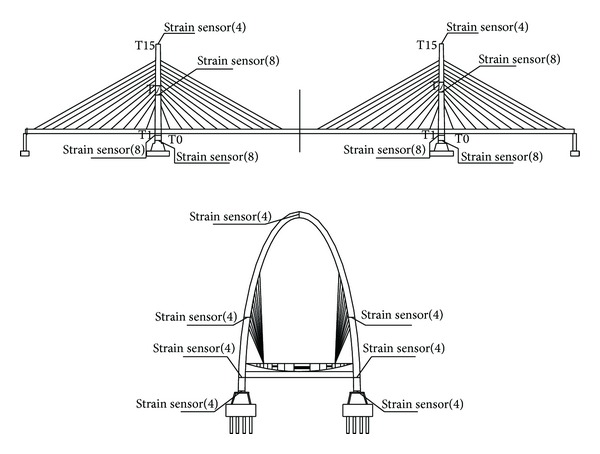
Stress and fatigue monitoring points on the steel tower.

*Stress, Fatigue, and Welding Crack Monitoring.* The high-stress sections on the steel box girders of the double-towered, cable-stayed bridges are generally present in the middle of each span, the juncture of a box girder and a cable tower, and the load-bearing position. The high-stress sections of the cable tower are generally present at the bottom of the cable tower and the juncture of the cable tower and steel box girder. Therefore, the sections in the middle of each span of the main bridge, the quarter-point of the main span, and the pier tops are used to monitor the steel box girder stress, fatigue, and welding cracks (Figures 8 and 9). As shown in Figure 10, the sections at T0, T1, and T7, the top of the tower, and the steel horizontal beam of the steel arch tower were selected to monitor stress and welding cracks in the steel arch tower.

**Figure 11:**
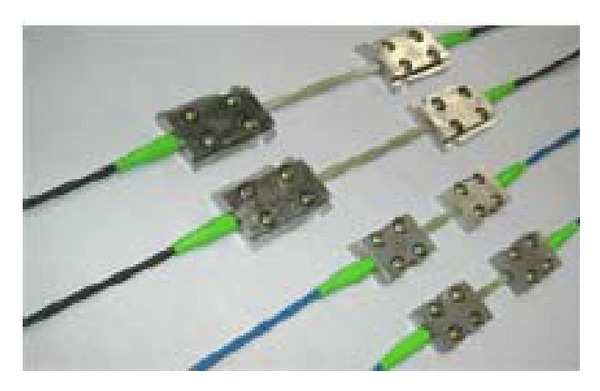
Strain gauges.

According to the analysis of the structure with different vehicle loads, the stress on the box girder top slab in the lateral direction is relatively high at the longitudinal diaphragm and that in the longitudinal direction is relatively high at the web. The monitoring points selected in this section were based on the layout of the bridge to ensure continuous monitoring. Strain gauges are used to monitor Strain (Figure 11), the number of sensors in each monitoring position is shown in Table 6 and the specifications are shown in Table 7.

*Structural Temperature Monitoring*. The main aims of structural temperature monitoring are to facilitate temperature compensation during stress monitoring and to determine the temperature ranges of key sections. Thus, the sections used for temperature monitoring are the same as those used for stress monitoring. Optical fiber grating temperature sensors are used for structural temperature monitoring, where the measurement range is −20°C to 70°C and the measurement precision is less than ±0.2°C. The positions and the number of monitoring points are shown in Table 8.

**Figure 12:**
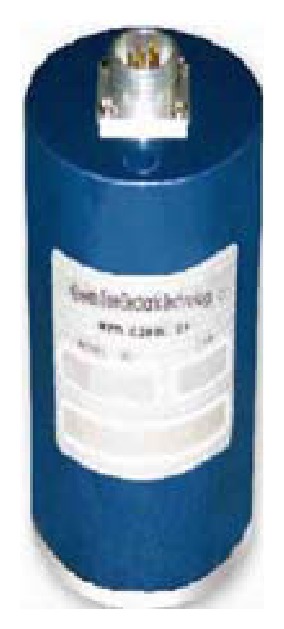
Vibration sensor.

*Structural Vibration Monitoring*. Structural vibration monitoring includes monitoring vibrations in the steel arch tower and the steel box girder, as well as the impact force on the bridge pier and earthquake responses. The vibration monitoring points on the steel box girder aim to ensure the maximum modal matrix type of the structural displacement. Therefore, the vibration sensors are positioned in the middle of each span, the quarter-point of the main span, and the pier tops on both sides of the main span of the steel box girder to monitor the vibrations of the steel box girder. To monitor the impact force on the bridge pier, the top of the pile cap of each navigable span is used as a vibration monitoring point. In addition, a vertical vibration sensor is placed at the same position to monitor the impact force and earthquake responses. The vibration sensor is shown in Figure 12 and the sensor layout is shown in Table 9.

Acceleration sensors are used to monitor vibrations of the steel arch tower and steel box girder, the impact force of the bridge pier, and earthquake responses, where the measurement range is ±2 g, the range of the frequency response is zero to 100 Hz, the dynamic range is >120 dB, and the working temperature range is from −20°C to 80°C.

*Cable Force Monitoring.* The cable is the main component of a cable-stayed bridge, which transfers the weight of the main girder and the live load on the deck to the main tower. The cable force is an important parameter of a cable-stayed bridge. Thus, it is important to enhance cable force monitoring to evaluate the working status of the cable and to analyze the stress state of a cable-stayed bridge. The amplitude value of cable must be controlled within a certain range to ensure the flexural strength of the cable, fatigue strength, and safety of users. Therefore, amplitude monitoring must form the basis of cable tension monitoring. The cables with the maximum stress under different load combinations and the maximum variation in stress under live loadings are used for cable tension monitoring.

**Figure 13:**
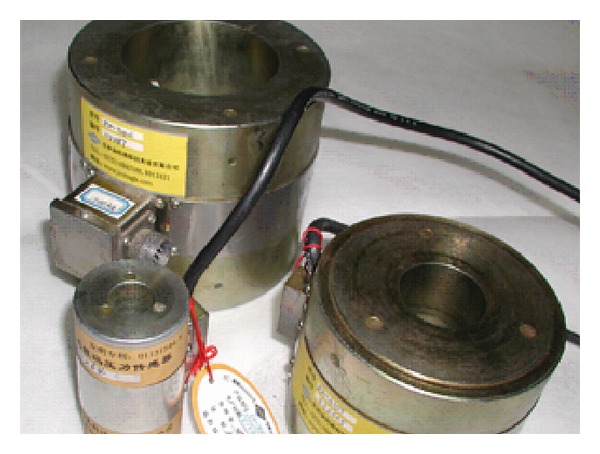
Acceleration sensor.

Acceleration sensors are used to monitor the tension of stayed cables (Figure 13), where the measurement range is ±10 g, the range of the frequency response is zero to 100 Hz, the dynamic range is >80 dB, and the work temperature is −20°C to 80°C. The positions and numbers of sensors are shown in Table 10.

*Anchor Force Monitoring*. In Zhijiang Bridge, the reliability of the juncture of the steel tower, concrete bearing, and foundation is the key to guaranteeing the structural safety of the whole bridge. This is because the static disequilibrium of the steel tower in a horizontal direction, the dynamic response to wind load and earthquake, and the connection between the steel tower and the bearing would be damaged by a major horizontal force and bending moment. Thus, the juncture of steel and concrete is the key to transmitting the force in a smooth and stable manner. An anchor is used to connect the main tower and the cap of Zhijiang Bridge. The pretightening force of the anchor directly reflects the operational status of the juncture segment of steel and concrete. Thus, it is necessary to monitor the variation in the anchor force in real-time.

To guarantee the stability and durability of anchor force monitoring at the juncture of steel and concrete, fiber grating strain sensors are used and the total number of monitoring points is 32.

#### 3.1.2. Data Acquisition and Transmission Module

The overall design of the data acquisition and transmission module comprises the components of the module and the functional flow of each component. The data preprocessing and temporary storage systems must meet the functional requirements of the data acquisition and transmission module. Thus, a low-level instrument must be installed in the field. The data acquisition and transmission module comprise an acquisition facility, transmission facility, and data preprocessing and temporary storage facility. The data transmission modes between the sensor and acquisition facility and between the acquisition facility and low-level instrument are wireless and wired transmission, respectively. To improve the stability of the data transmission in the SHM system, wired data transmission is used between the sensor and acquisition facility, and between the acquisition facility and the low-level instrument. Monitoring points are present all over the main bridge and the approach near the main bridge, so the transmission distance from some far-end sensors to the low-level instrument would be very long if only one low-level instrument was used, which would affect the efficiency and quality of data transmission. Therefore, two low-level instruments are positioned in the steel box girder section at the junctures of two steel arch towers and the steel box girders on the main bridge. Data are transferred between the low-level instruments and the monitoring center via an embedded optical communication cable.

**Figure 14:**
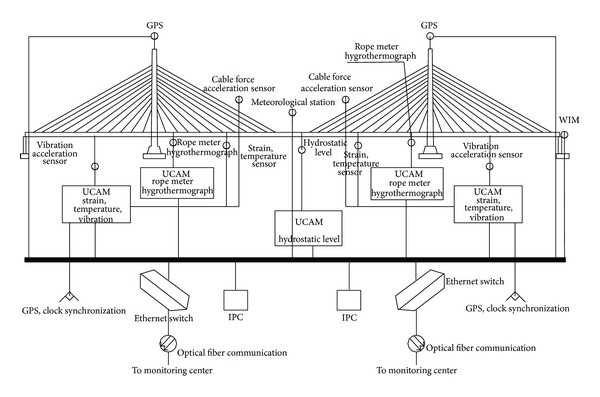
Flowchart of data acquisition and transmission.

Figure 14 shows the function flow during data acquisition and transmission. All of the different types of sensors are connected via an anti-interference shielding line and the corresponding acquisition facility. All of the acquisition facilities are connected to an industrial control computer via LAN and an Ethernet switch, which comprise the field data acquisition and transmission network. The Ethernet switch is also connected to an Ethernet switch in the bridge monitoring center via optical fiber communication wires and cables, which forms a data transmission network between the bridge and monitoring center. The relationship between the industrial control computer and the ethernet switch is a one-to-one correspondence, where two groups are present in the steel box girder section under the two main towers of the bridge. The acquisition facilities for stress, temperature, and vibration are integrated into the steel box girder section under the two main towers of the bridge. The transmission distance is longer than 200 m, so to guarantee highly synchronized vibration acquisition, the GPS clock is used for clock synchronization between the two acquisition stations for the stress, temperature, and vibration sensors.

#### 3.1.3. Software System

**Figure 15:**
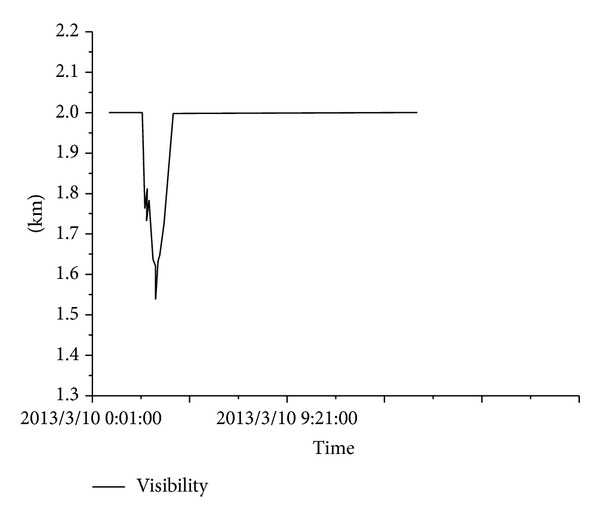
External environment of the bridge.

**Figure 16:**
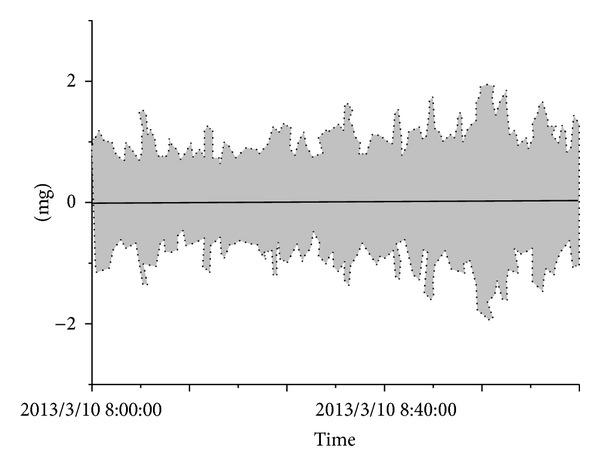
Vibration of 4# tower and T7 box girder.

**Figure 17:**
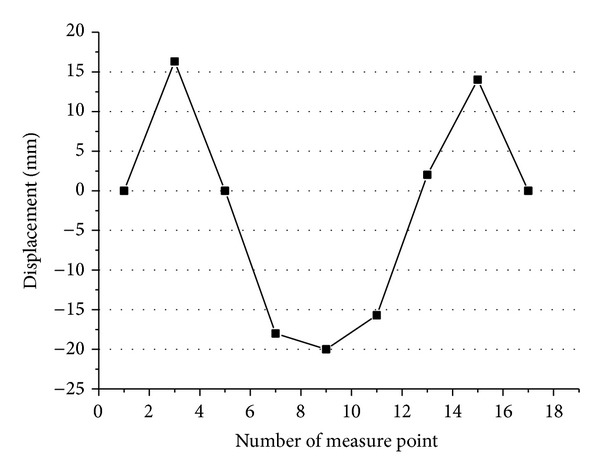
Displacement changes in the box girder.

**Figure 18:**
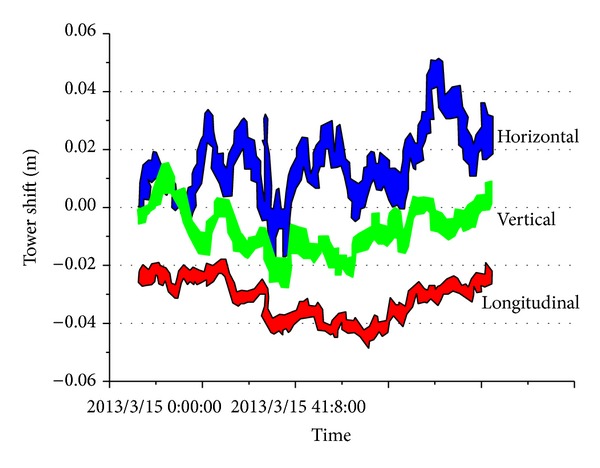
Tower shift.

**Figure 19:**
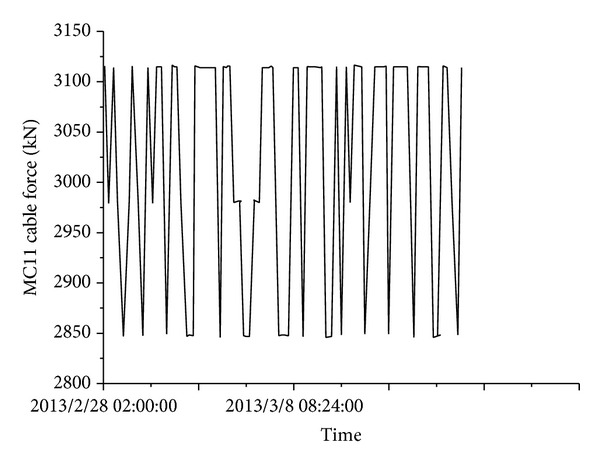
Cable force.

The design of the system software must meet the needs for data acquisition and transmission control, maintenance checking data management, data comprehensive management, data analysis and status evaluation, maintenance decision and safety prealarming, and user management. According to the requirements for function monitoring, the software is divided into data acquisition and transmission software, maintenance management software, center database software, structural status evaluation software, maintenance decision and safety prealarming software, user interface software, and other components. The data acquisition and transmission software includes an acquisition instruction module, parameter setting module, data preprocessing module, abnormal event log module, software self-repair module, graph display module, and other components. All of the hardware and software in the SHM system include auto-command and auto-call functions for the software. The system runs on a network and some examples of the results obtained are shown in Figures 15, 16, 17, 18, and 19.

### 3.2. Design and Implementation of the DMS

**Figure 20:**
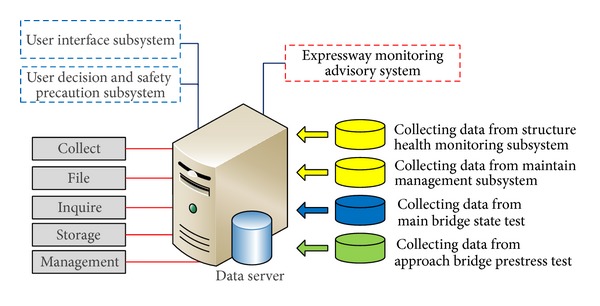
Function and composition of the DMS.

The DMS (Figure 20) used by the SHM system of Zhijiang Bridge includes a data management subsystem; the function of which is to acquire data from the health monitoring subsystem, maintain data from the maintenance management subsystem, monitor data from the road floor status, and collect, file, inquire, store, and manage the monitored prestress data.

### 3.3. Design and Implementation of the EDS

The structural status evaluation subsystem is the core of the integrated system. The EDS subsystem executes various operations such as calculation analysis and it generates statistics for monitoring data, historical alignment, analysis and trend forecasting for monitoring data, and fetches the key indices that reflect the variations in the structural status. Based on an analysis of the structure, the variation in each status index, maintenance checking data, and other scientific research measurements, the overall data are integrated to facilitate a comprehensive evaluation of the status of the bridge structure and its key components.

The structural status evaluation subsystem is divided into the data processing module and the status evaluation module. The main functions of these modules are as follows.

1. The data processing module is responsible for filtering, classification, collection, and statistical analysis of the data, and for fetching the key indices.
2. The status evaluation module is responsible for real-time analysis and evaluating the structural status.

#### 3.3.1. Data Processing Module

The volume of bridge monitoring data is massive, so they must be finely analyzed in detail to obtain useful key information. The functions of the data processing module are as follows.

1. The original test values from each sensor are integrated to obtain the primary status of the bridge health monitoring data.
2. Filtering, classification, collection, and statistical analyses of the monitoring data are performed to obtain an eigenvalue database of the monitoring data.
3. Based on the monitoring results and comparisons with the values in the design document, the experimental bridge loading values, modified values from simulation calculations, and the extreme values during the operational process, the variations in the rates of the values that reflect the structural status can be obtained, which can be analyzed to identify trends using mathematical model fitting.
4. The degree of deviation and rate of development are calculated based on comparisons with the threshold values of all the bridge safety prealarming levels.

#### 3.3.2. Status Evaluation Module

**Figure 21:**
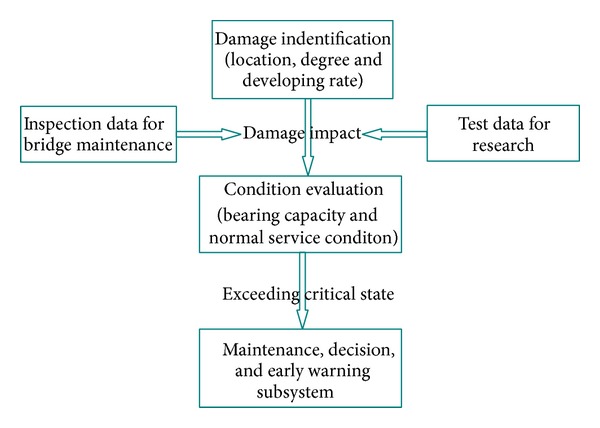
Flowchart during bridge condition evaluation.

The core functions of the status evaluation module are damage identification and status evaluation. The work flow of the monitoring system first identifies the position, degree, and rate of development of structural damage based on abnormal data, before comprehensively evaluating the effects of damage on the structure by combining the bridge maintenance checking data with the scientific monitoring data, thereby confirming the user status of the bridge. If the user state exceeds the critical condition, the evaluation information is passed to the maintenance decision and safety prealarming subsystem. The flow during status evaluation is shown in Figure 21.

Many methods are suitable for damage identification and structural evaluation, and each method has specific characteristics and appropriate conditions. Thus, we selected methods that satisfy the structural type, materials, and work environment of Zhijiang Bridge. Methods such as module modification, structural fatigue analysis, primary fingerprint comparison, trends analysis, model state analysis, parameter identification, and reliability evaluation are used to localize, quantify, and identify damage by monitoring trends and evaluating their effects.

### 3.4. Design and Implementation of the ASS

The ASS used by the SHM system of Zhijiang Bridge comprises a user interface subsystem, which is an interactive system for bridge maintenance management users. The functions implemented in the subsystem include a monitoring point information display, monitoring results graphical display, report management, background management, and user management. The comprehensive management software implemented in this system provides many functions, such as information management and querying of monitoring points, monitoring and maintenance of data queries, graphical displays, state evaluation result querying, report management and inquiry, prealarming information management and inquiry, background management, and user management.

## 4. Conclusions

The core problems and final aims of SHM systems for cable-stayed bridges are damage identification, module modification, structural safety evaluation, and maintenance decision making. The implementation and operation of the various subsystems and the overall system facilitate these aims. In the present study, we used the integrated system of structural health monitoring and intelligent management of Zhijiang Bridge as an example to provide a detailed explanation of the components and model functions employed by a large-scale bridge SHM system. This SHM system generates time-specific status information such as bridge vibrations, thereby providing data support for bridge maintenance and decision making, which reduces the maintenance cost and improves the technical level of long-term management. The application of SHM systems to large-scale Chinese bridges is in the early stage. Thus, the appearance of new technology and new methods will be beneficial to improving SHM systems. In particular, wireless communication technology such as microwaves may be an important method in SHM system networks for bridge structures, which could be applied broadly to health monitoring and the measurement of bridge structures.

## Figures and Tables

**Table 1:** Meteorological station technical specifications.

Parameter	Technical specifications
Wind speed	Measurement range: 0–60 m/s; resolution: 0.1 m/s; precision: ±0.3 m/s
Wind direction	Measurement range: 0–359°; resolution: 1°; measurement accuracy: < ±3°
Temperature	Measurement range: −20°C to 70°C; measurement accuracy: < ±0.2°C
Relative humidity	Measurement range: 0–100% RH; measurement accuracy: < ±2% RH
Rainfall	Measurable raindrop size range: 0.3–5 mm; resolution: 0.01 mm
Visibility	Measurement range: 10–20000 m; resolution: 0.1 m; precision: +2%

**Table 2:** Hygrothermograph technical specifications.

Project	Technical specifications
Temperature	Measurement range: −20°C to 70°C; measurement accuracy: < ±0.2°C

Relative humidity	Measurement range: 0–100% RH; measurement accuracy: < ±2% RH

**Table 3:** Weigh-in-Motion system technical specifications.

Parameter	Technical specifications
Speed range	0~180 km/h
Traffic counting accuracy	±0.1%
Average speed accuracy	±1.5%
Gross vehicle weight accuracy	±3%

**Table 4:** Technical specifications of the GPS sensors.

Parameter	Technical specifications
Static long baseline solution precision	Horizontal: 3 mm + 0.5 ppm
	Vertical: 6 mm + 1 ppm
Fast static baseline solution precision	Horizontal: 5 mm + 0.5 ppm
	Vertical: 10 mm + 1 ppm
Dynamic point baseline solution precision	Horizontal: 10 mm + 1 ppm
	Vertical: 20 mm + 2 ppm
Control function	Real-time RTK function, with a maximum sampling frequency of ≥5 Hz
Transmission performance	Real-time automatic collection, 24 h without exception
Working environment	Receiver and terminal: −20°C to 50°C; antenna: −40°C to 50°C

**Table 5:** Technical specifications of the hydrostatic level gauge.

Parameter	Technical specifications
Measurement range	±300 mm
Measurement accuracy	±0.5% FS
Sensitivity	0.05 FS
Operating temperature	−20°C to 80°C
Protection class	IP67

**Table 6:** Strain sensor arrangement.

Monitoring position	Number
Midsection of the main span	12
1/4, 3/4 section of main span	24
Midsection of the side span	24
Section of steel box girder at each pier top	16
T0, T1, and T7 and top section of steel arch tower	56
Section of the lateral steel beam	16
Section of the approach	100

**Table 7:** Technical specifications of the strain sensor.

Parameter	Technical specifications
Measurement range	±2000 *μ* *ε*
Resolution	±1 *μ* *ε*
Measurement accuracy	±2-3 *μ* *ε*
Strain sensitivity	1.18–1.22 pm/*μ* *ε*

**Table 8:** Position and number of temperature monitoring points.

Monitoring position	Sensor type	Number
Midsection of the main span	Temperature sensor	2
1/4, 3/4 section of main span	Temperature sensor	4
Midsection of the side span	Temperature sensor	4
Section of the steel box girder at each pier top	Temperature sensor	8
T0, T1, and T7, and top section of steel arch tower	Temperature sensor	32
Section of the lateral steel beam	Temperature sensor	4
Section of the approach	Temperature sensor	14

**Table 9:** Layout of the vibration sensors.

Monitoring position	Sensor type	Number
T0, T7, and top section of tower	Vibration sensor	10

Top of the pile cap of each navigable span	Vibration sensor	8

1/4, 1/2 section of the main-span, 1/2 section of the side-span, and section of the pier top	Vibration sensor	8

**Table 10:** Positions and numbers of acceleration sensors.

Monitoring positions	Number
1, 1, 11# cables in the side-spans of the east and west sides	12
1, 7, 11# cables in the main-span of the east and west sides	12
